# Digital X-ray radiogrammetry of hand or wrist radiographs can predict hip fracture risk—a study in 5,420 women and 2,837 men

**DOI:** 10.1007/s00330-012-2706-9

**Published:** 2012-11-16

**Authors:** M. L. Wilczek, J. Kälvesten, J. Algulin, O. Beiki, T. B. Brismar

**Affiliations:** 1 Karolinska Institutet, Department for Clinical Science, Intervention and Technology, Division of Radiology, Karolinska University Hospital, Stockholm, Sweden; 2 Sectra Imtec AB, Linköping, Sweden; 3 Centre for Medical Image Science and Visualization (CMIV), Linköping University, Linköping, Sweden; 4 Department of Medicine Solna, Unit of Clinical Epidemiology, Karolinska Institutet, Stockholm, Sweden; 5 Kermanshah University of Medical Sciences, Kermanshah, Islamic Republic of Iran; 6 Frejgatan 34, 113 26 Stockholm, Sweden

**Keywords:** Osteoporosis, Hip fracture, Digital X-ray radiogrammetry, Cohort, BMD

## Abstract

**Objectives:**

To assess whether digital X-ray radiogrammetry (DXR) analysis of standard clinical hand or wrist radiographs obtained at emergency hospitals can predict hip fracture risk.

**Methods:**

A total of 45,538 radiographs depicting the left hand were gathered from three emergency hospitals in Stockholm, Sweden. Radiographs with insufficiently included metacarpal bone, fractures in measurement regions, foreign material or unacceptable positioning were manually excluded. A total of 18,824 radiographs from 15,072 patients were analysed with DXR, yielding a calculated BMD equivalent (DXR-BMD). Patients were matched with the national death and inpatient registers. Inclusion criteria were age ≥ 40 years, no prior hip fracture and observation time > 7 days. Hip fractures were identified via ICD-10 codes. Age-adjusted hazard ratio per standard deviation (HR/SD) was calculated using Cox regression.

**Results:**

8,257 patients (65.6 % female, 34.4 % male) met the inclusion criteria. One hundred twenty-two patients suffered a hip fracture after their radiograph. The fracture group had a significantly lower DXR-BMD than the non-fracture group when adjusted for age. The HR/SD for hip fracture was 2.52 and 2.08 in women and men respectively. The area under the curve was 0.89 in women and 0.84 in men.

**Conclusions:**

DXR analysis of wrist and hand radiographs obtained at emergency hospitals predicts hip fracture risk in women and men.

**Key Points:**

• *Digital X-ray radiogrammetry of emergency hand/wrist radiographs predicts hip fracture risk.*

• *Digital X-ray radiogrammetry (DXR) predicts hip fracture risk in both women and men.*

• *Osteoporosis can potentially be identified in patients with suspected wrist fractures.*

• *DXR can potentially be used for selective osteoporosis screening.*

## Introduction

According to epidemiological studies, about one third of women over 50 years of age will experience a fragility fracture [1, 2]. The number of fractures can be reduced by adequate measures, such as lifestyle adaptations [3–6] and pharmaceutical interventions [7–10]. To be cost effective and avoid unnecessary and potentially harmful pharmaceutical treatment, it is imperative that those at risk are properly identified and targeted.

Previous studies have shown a correlation between bone mineral density (BMD) and the risk of fracture [11–15]. Consequently, BMD measurement has a prominent position in fracture risk assessment and diagnosis of osteoporosis. Dual-energy X-ray absorptiometry (DXA) of the hip or spine is considered the gold standard [12, 15, 16]. Unfortunately, most patients with a high risk of osteoporosis are not scanned by DXA, even when the clinician suspects osteoporosis. This is due to the relatively high costs [17, 18] and low availability of equipment [19–22].

**Fig. 1:**
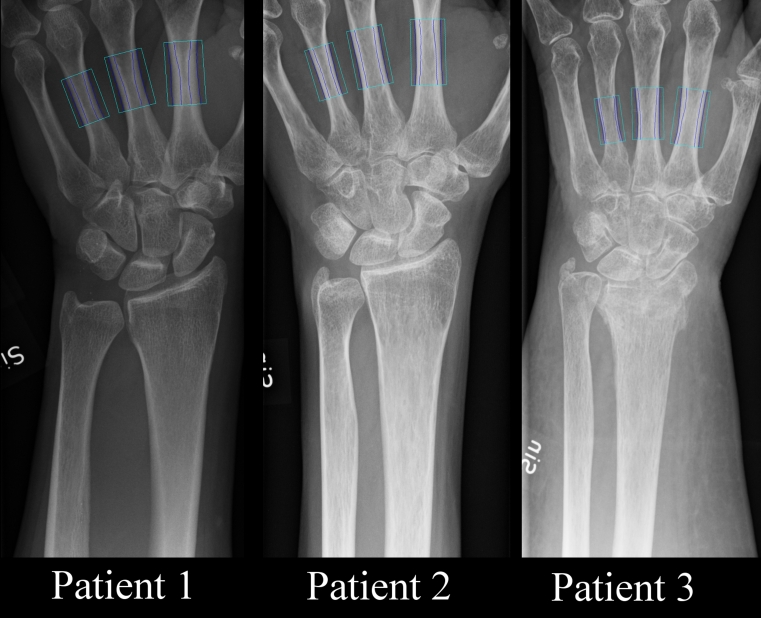
Wrist radiographs used for DXR analysis of patients with varying DXR T-scores. Measurement regions are indicated over metacarpals II-IV. Patient 1: a 50-year-old woman. DXR T-score 1.18. DXR-BMD 0.64 g/cm^2^. Patient 2: a 48-year-old woman. DXR T-score −1.90. DXR-BMD 0.49 g/cm^2^. Patient 3: a 75-year-old woman. DXR T-score −3.70. DXR-BMD 0.41 g/cm^2^

Digital X-ray radiogrammetry (DXR) is a peripheral measurement method based on a combination of an average geometrical measure of the cortical thickness of metacarpals II-IV and structural analysis of cortical bone porosity [23, 24]. The analysis is based on a standard radiograph of the hand (Fig. 1) and gives a computed BMD equivalent measurement. This makes DXR an interesting and potentially cost-effective candidate for the evaluation of osteoporosis-related fracture risk.

The objective of this study was to assess whether DXR analysis can predict hip fracture risk by using standard clinical hand or wrist radiographs obtained at emergency hospitals.

## Materials and methods

This cohort study includes data from 1 January 2000 to 31 December 2008. The data were retrieved in 2009 and 2010. The ethical committee in Stockholm approved the study.

### Radiograph selection

Database queries based on examination codes were used to extract all left hand and wrist radiographs from the digital archives of three major emergency hospitals in the Stockholm region. Clinical indications for left hand or wrist radiographs included suspicion of fracture, luxation, foreign body or arthritis.

**Fig. 2:**
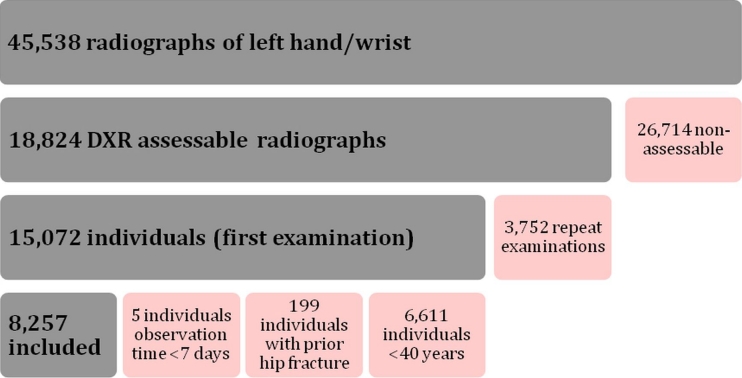
Flow chart of how radiographs and patients were selected (*gray* = inclusion, *red* = exclusion)

A total of 45,538 radiographs were extracted from the digital archives. All measurements and radiographs were manually reviewed by two of the investigators, who were blinded to all clinical information, and assessed according to suitability for further DXR analysis. Inclusion criteria were radiographs of the left hand or wrist depicting sufficient metacarpal bone for DXR analysis. Exclusion criteria were fractures in the measurement regions, foreign material such as fixation pins and plaster, or unacceptable positioning of the metacarpals. In all, 18,824 examinations from 15,072 patients were considered suitable for DXR analysis. In patients with repeated examinations, only the chronologically first evaluable examination was used. The image and patient selection process is illustrated in a flow chart (Fig. 2).

### Patient selection

All patients whose radiographs were included for DXR analysis were identified in the National Patient Register provided by the National Board of Health and Welfare. Patients who subsequently had a hip fracture were identified via ICD-10 codes (S72.0, S72.1, S72.2). Inclusion criteria were age >40 years, no hip fracture prior to acquisition of the radiograph and observation time >7 days. To minimise the risk of erroneous registrations, only those coded for both diagnosis and adequate intervention (either upper femur fracture surgery or hip replacement, i.e. ICD-10 surgical codes NFJ and NFB) were registered as hip fracture. In order not to exclude patients with a hip fracture who were too critically ill for surgery, patients who died within 3 days after a registered fracture were also included. Date of fracture diagnosis, date of death or study end date (31 December 2008) was used as censoring time. The National Cause of Death Register provided date of death.

### Digital X-ray radiogrammetry and BMD

Digital X-ray radiogrammetry (DXR) (Onescreen, Sectra Imtec AB, Linköping, Sweden) is a development of the traditional technique of radiogrammetry. On a standard projection radiograph, measurement regions are automatically placed around the narrowest parts of metacarpals II-IV. A BMD equivalent measurement (DXR-BMD) is then computed. The calculation is defined as *DXR-BMD = cxVPAx(1-p)* where *c* is a density constant empirically determined so that DXR-BMD on average is equal to the mid-distal forearm region of the Hologic QDR-2000 densitometer (Hologic, Bedford, MA, USA), *VPA* is cortical bone volume per area and *p* is porosity. When comparing an individual’s DXR-BMD to the mean DXR-BMD of a young, healthy, normal reference population, a DXR T-score can be derived. When compared to a healthy reference population of the same age, a DXR Z-score is obtained.

Any digital or CR radiography equipment that is applicable for acquiring hand X-ray images can be used to acquire images for DXR-BMD analysis. The DXR analysis process is automated and operator independent. However, there are some requirements about positioning and exposure settings, e.g., when acquiring radiographs intended for DXR analysis. Some requirements are generic (posterior-anterior X-ray image of one hand, palm flat to detector table/image plate surface, focus centred on metacarpal III) and some are specific per modality type and model (image postprocessing settings, focus distance, exposure settings, location on detector).

The DXR technology has been described in more detail previously [23, 24], and normative reference tables have been published [25, 26].

### Statistical analysis

**Fig. 3:**
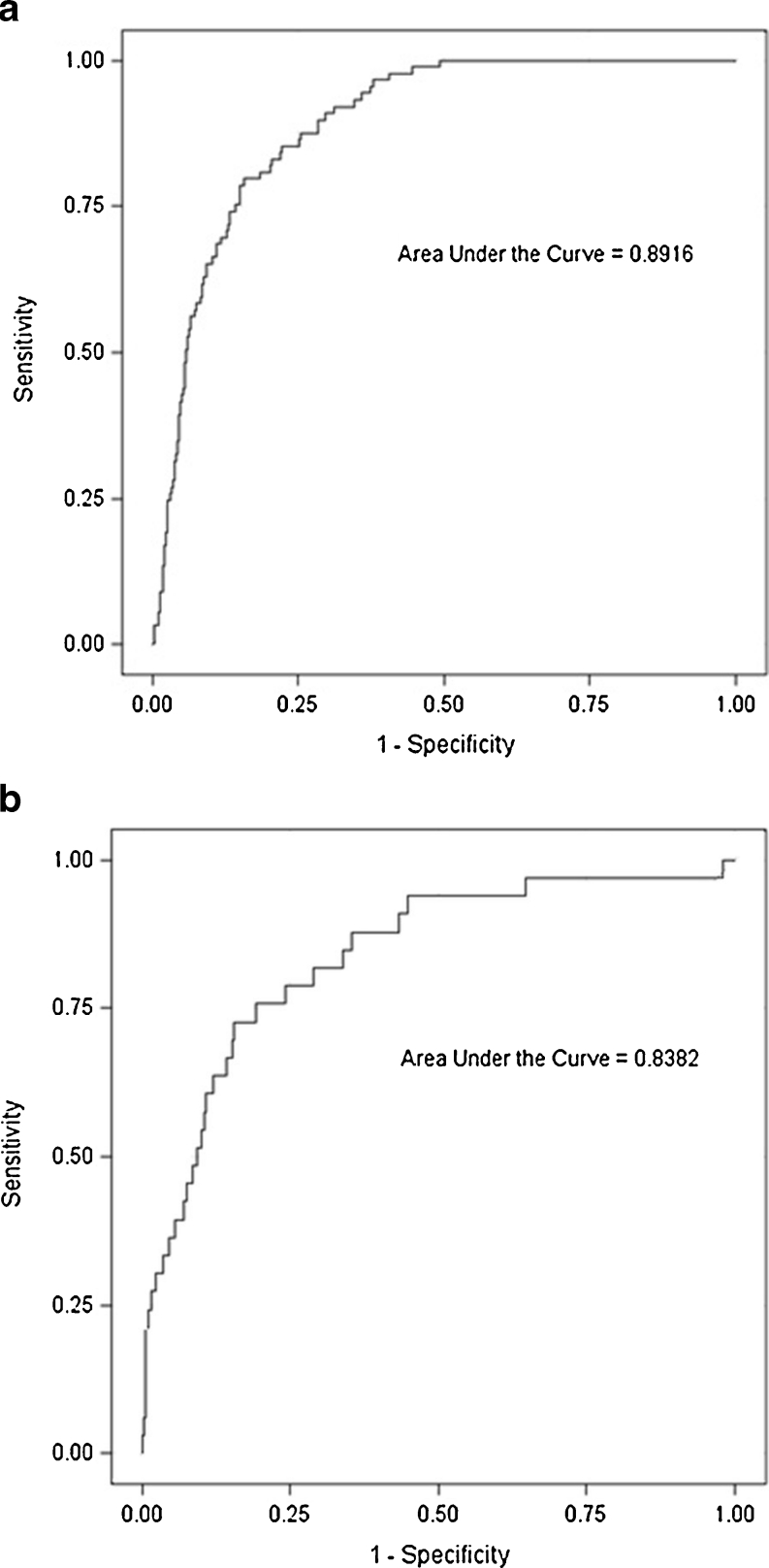
**a** Age-adjusted ROC curve for women. **b** Age-adjusted ROC curve for men

Group comparisons were made using Student’s t-test for continuous normally distributed data. Receiver-operator characteristics (ROC) were plotted (Fig. 3) to evaluate the predictive value of the DXR T-score to assess fracture risk. In these graphs the sensitivity of a parameter, in this case DXR T-score, to predict future fractures is plotted as a function of the proportion of false positives (1-specificity). To compensate for age-related fracture risk that is not related to DXR-BMD (e.g. the increased tendency to fall), the ROCs were adjusted for age. To enable comparison among different studies, the area under the plotted age-adjusted ROC curve, the AUC and the age-adjusted hazard ratio per standard deviation change in DXR T-score (HR/SD) were calculated. The HR/SD was calculated using Cox regression and the risk of sustaining a hip fracture at DXR T-scores >−1 was defined as risk = 1. SAS® 9.2 (SAS Institute, Cary, NC) was used for the statistical analysis.

## Results

The inclusion criteria were met by 8,257 patients (65.6 % women; 34.4 % men). The average age was 59.6 years (SD 12) (60.5 years in women; 57.8 years in men). The average follow-up period was 3 years 3 months with a total observation time of 27,072 person-years. The number of patients, average DXR-BMD, DXR T-score, number of fractures and annual fracture rate are provided per 5-year age group in Table 1 and per DXR T-score in Table 2.

**Table 1:** Distribution of numbers, bone mineral density (DXR-BMD), DXR T-score and fracture rate by 5-year age groups

Age	Number		Mean DXR-BMD (standard deviation)		Mean DXR T-score (standard deviation)		Fractures		Mean annual fracture rate (%)	
	Female	Male	Female	Male	Female	Male	Female	Male	Female	Male
40–44	523	472	0.59 (0.05)	0.66 (0.06)	0.19 (1.14)	−0.20 (1.01)	0	1	0.00	0.07
45–49	598	424	0.58 (0.05)	0.66 (0.06)	0.01 (1.08)	−0.27 (1.02)	1	2	0.05	0.14
50–54	747	404	0.56 (0.05)	0.65 (0.06)	−0.43 (1.15)	−0.41 (1.02)	1	3	0.04	0.23
55–59	982	426	0.55 (0.05)	0.64 (0.06)	−0.78 (1.14)	−0.53 (0.99)	2	4	0.06	0.27
60–64	847	360	0.52 (0.06)	0.63 (0.06)	−1.33 (1.18)	−0.69 (0.97)	8	5	0.29	0.45
65–69	558	239	0.50 (0.06)	0.60 (0.06)	−1.80 (1.27)	−1.11 (1.01)	8	0	0.44	0.00
70–74	403	192	0.47 (0.06)	0.59 (0.07)	−2.35 (1.21)	−1.39 (1.10)	9	2	0.67	0.35
75–79	338	154	0.44 (0.06)	0.58 (0.07)	−2.93 (1.18)	−1.54 (1.11)	17	4	1.47	0.92
80–84	260	104	0.43 (0.06)	0.56 (0.07)	−3.24 (1.16)	−1.91 (1.17)	18	5	2.19	1.63
85–89	116	48	0.41 (0.05)	0.53 (0.06)	−3.74 (1.00)	−2.37 (0.98)	18	4	6.90	3.78
90–94	38	12	0.39 (0.05)	0.48 (0.07)	−4.10 (1.05)	−3.06 (1.16)	7	2	9.83	14.63
95–99	10	2	0.37 (0.04)	0.49 (0.05)	−4.49 (0.85)	−2.93 (0.88)	0	1	0.00	98.98
Total	5420	2837	0.53 (0.08)	0.63 (0.07)	−1.20 (1.60)	−0.69 (1.16)	89	33	0.49	0.37

**Table 2:** Distribution of numbers, observation time and fracture rate by various DXR T-scores

DXR T-score	Number	Mean age	SD age	Fractures	Observation years	Annual fracture rate (%)
Female						
<−6	5	74.9	12.0	1	18	5.71
−6 < −5	44	83.5	8.8	5	121	4.12
−5 < −4	235	78.7	8.9	23	698	3.30
−4 < −3	516	74.2	9.4	40	1,573	2.54
−3 < −2	803	66.9	9.6	10	2,634	0.38
−2 < −1	1,172	61.0	9.3	6	3,834	0.16
−1 < 0	1,376	55.9	8.3	4	4,733	0.08
0 < 1	870	52.1	7.5	0	3,006	0.00
1 < 2	336	49.9	7.1	0	1,238	0.00
2 < 3	57	47.4	11.0	0	211	0.00
3 < 4	4	46.7	7.5	0	10	0.00
4 < 5	2	51.5	11.5	0	8	0.00
Total	5,420	60.5	12.0	89	18,083	0.49
<−2.5	1,165	73.6	10.2	72	3,581	2.01
Male						
<−6	1	81.0	–	1	5	19.50
−6 < −5	–	–	–	–	–	–
−5 < −4	16	74.6	11.3	0	42	0.00
−4 < −3	81	74.1	12.8	8	217	3.68
−3 < −2	264	67.7	12.9	9	768	1.17
−2 < −1	679	61.0	12.3	9	2,070	0.43
−1 < 0	1,011	55.8	10.6	4	3,296	0.12
0 < 1	610	53.1	9.1	1	1,959	0.05
1 < 2	157	49.9	8.2	1	562	0.18
2 < 3	18	46.7	5.2	0	69	0.00
3 < 4	–	–	–	–	–	–
4 < 5	–	–	–	–	–	–
Total	2,837	57.8	12.2	33	8,989	0.37
<−2.5	199	72.1	12.5	12	553	2.17

During the observation period, 122 patients (89 women and 33 men) suffered from a hip fracture. In both men and women, the patients who suffered from a hip fracture were significantly older at the time of X-ray examination and had a significantly lower DXR-BMD, DXR T-score and DXR Z-score than those who did not (Table 3). Out of the 122 patients who suffered a hip fracture, 84 (72 women and 12 men, 69 %) had a DXR T-score of  ≤−2.5, resulting in a sensitivity of 81 % and a specificity of 79 % in women and 36 % and 93 % respectively in men (Table 4).

**Table 3:** Differences between fracture and non-fracture groups by sex. One standard deviation is given within parentheses. All differences were statistically significant at the *P* < 0.001 level

	Number	Age at exam (years)	DXR-BMD (g/cm^2^)	DXR T-score	DXR Z-score
Female					
Fracture	89	78 (10)	0.419 (0.05)	−3.5 (1.1)	−0.565 (0.988)
Non-fracture	5,331	60 (12)	0.528 (0.08)	−1.2 (1.6)	0.009 (0.997)
Male					
Fracture	33	70 (16)	0.538 (0.09)	−2.2 (1.4)	−0.900 (1.110)
Non-fracture	2,804	58 (12)	0.630 (0.07)	−0.7 (1.1)	0.030 (0.994)

**Table 4:** The sensitivity and specificity of DXR to predict hip fracture at DXR T-score ≤−2.5, with 95 % confidence intervals and the corresponding annual fracture rate

	Age (years)	Sensitivity (95 % CI)	Specificity (95 % CI)	Annual fracture rate (%)
Women	>40	81 (73–89)	79 (78–80)	2.01
	55–85	76 (66–86)	72 (70–73)	1.62
Men	>40	36 (20–53)	93 (92–94)	2.17
	55–85	25 (6–44)	90 (89–92)	1.18

The age-adjusted AUC for the DXR T-score to predict hip fracture was 0.89 in women and 0.84 in men (Fig. 3). In the 55–85-year age group, the corresponding AUCs were 0.83 and 0.80, respectively.

**Fig. 4:**
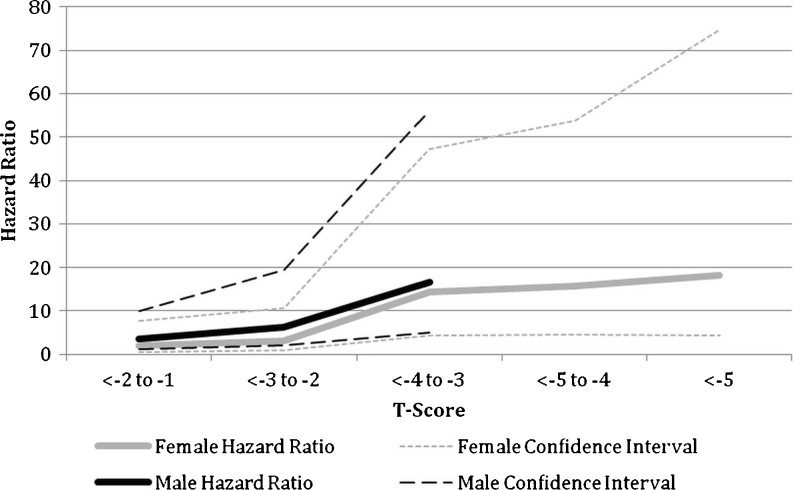
Hazard ratio by various DXR T-scores for female and male subjects. No data shown for men at DXR T-score <−4 because of insufficient numbers of subjects

Age-adjusted hazard ratios for hip fracture at different DXR T-scores are shown in Fig. 4 and Table 5. The hazard ratio per standard deviation change in DXR T-score (HR/SD) for hip fracture was 2.52 and 2.08 in the female and male group, respectively. In the 55–85-year age group, the corresponding values were 2.33 and 2.00 for women and men, respectively.

**Table 5:** Age-adjusted hazard ratios for hip fracture for different DXR T-scores by sex. DXR T-score >−1.0 was defined as HR = 1

DXR T-score	Hazard ratio	95 % Confidence interval		Number	Fractures	Observation years
Female						
<−2 to −1	2.1	0.56	7.7	1,172	6	3,834
<−3 to −2	3.1	0.87	10.8	803	10	2,634
<−4 to −3	14.4	4.42	47.2	516	40	1,573
<−5 to −4	15.7	4.59	53.8	235	23	698
<−5	18.3	4.45	74.8	49	6	139
Male						
<−2 to −1	3.5	1.20	10.1	679	9	2,070
<−3 to −2	6.3	2.01	19.6	264	9	768
<−4 to −3	16.7	4.99	56.1	81	8	217
<−5 to −4	0.00	0.00	–	16	0	42
<−5	42.3	4.30	416.4	1	1	5

## Discussion

In this study, DXR-BMD of archived radiographs of the wrist or hand were used to evaluate the association with hip fractures occurring after the radiograph had been obtained. In order to evaluate whether DXR of radiographs of the wrist and hand obtained at emergency hospitals can be used for selected screening for osteoporosis, so-called area under the curve (AUC) calculations were made. Those describe the relationship between the sensitivity and specificity. Ideally such curves are close to 1, where only those who will suffer from a hip fracture are discriminated from those who will not. In this study, the age-adjusted AUC for the DXR T-score to predict hip fracture was 0.89 in women and 0.84 in men. These values are similar to, or higher than, those previously published for central DXA [14]. Bousson et al. compared quantitative computed tomography (QCT) and hip DXA in a female study population with a similar age distribution to our cohort and obtained AUCs almost as large as ours (0.84 for QCT and 0.80 for DXA) [27]. In the EPIDOS cohort, AUC calculations for hip fracture risk based on central DXA measurements ranged from 0.68 to 0.72 depending on the region measured [15]. However, the EPIDOS population was older and the age distribution smaller (average age 80.5, SD 3.8), which results in smaller AUCs. Cummings’ study, based on a younger population (average age 73.2) than the EPIDOS cohort, provided a slightly greater AUC, with a maximum of 0.78 for women undergoing DXA of Ward’s triangle (AUC was 0.76 when measured at the femoral neck) [28].

A meta-analysis from 1996 estimated that the increased risk of hip fracture with a decrease of 1.0 in T-score is 2.6 for hip BMD and 1.9 for lumbar spine BMD in women [29]. Later studies have shown similar results [13, 30]. When using DXR, Bouxsein et al. reported a hazard ratio per decrease of 1.0 in DXR T-score of 1.8 for hip fractures in a case-cohort study of a sample from the Study of Osteoporotic Fracture (SOF) cohort, i.e., community-dwelling US women aged >65 years [31]. In their prospective study DXR compared well to other peripheral BMD measurements, but was inferior to central BMD measurements when predicting hip fracture risk. In a DXR analysis of archived radiographs from a subgroup of the Copenhagen City Heart Study cohort, consisting of women >55 years with self-reported joint or bone pain, Bach-Mortensen et al. reported a HR/SD of 1.4 for hip fractures (*P* = 0.052) [32].

In our study the HR/SD for DXR in women aged 55–85 was 2.33 and in all women 2.52. Thus, DXR provided predictive values comparable to those previously published for central BMD measurements [29] and considerably higher than those previously reported for DXR [31, 32]. However, our study differs from the previous studies by Bouxsein’s and Bach-Mortensen’s groups with regard to the study population. Their studies included patients recruited from selected cohorts (SOF cohort and Copenhagen City Heart Study cohort, respectively). Our study reflects all patients undergoing standard X-ray examination of the left hand at three emergency hospitals in Stockholm. These groups might differ in terms of fracture and disease prevalence. The hazard ratio was also high in the male group; 2.08 in all men and 2.00 in men aged 55–85. To our knowledge, DXR’s predictability has not previously been studied in men.

Due to the inclusion of all left hand/wrist radiographs obtained at three emergency hospitals, the patients included were most likely those with a suspected wrist fracture, i.e. trauma, and patients undergoing routine control for rheumatoid arthritis. Distal forearm fractures have been shown to be associated with other fragility fractures and have therefore been suggested as a possible predictor in fracture risk assessment [12, 33–35]. The correlation between wrist fracture and other fragility fractures appears to be stronger in men [36]. Rheumatoid arthritis has been shown to increase the risk of fracture significantly [30, 37, 38]. Hence, the patients in our study were most likely at higher risk of fragility fractures than a normal population. This might also explain the improved performance of DXR observed in our study compared to previous reports [31, 32].

### Defining clinical thresholds

There are no previous recommendations on when patients should receive treatment based on DXR measurements. According to the National Osteoporosis Foundation clinician’s guide to prevention and treatment of osteoporosis, health-care providers should consider FDA-approved medical therapies in postmenopausal women and men aged 50 years and older at T-score ≤−2.5 at the femoral neck or spine or in the case of low bone mass (T-score between −1.0 and −2.5 at the femoral neck or spine) and a 10-year probability of a hip fracture ≥3 % [39]. In clinical fracture outcome trials, a T-score of −2.5 is one of the most commonly used inclusion criteria, for example in the FREEDOM trial [40]. A combination of low BMD and prevalent vertebral fracture has also been used for inclusion. In the HORIZON trial subjects could be included if they had femoral neck BMD with a T-score of ≤−2.5 or if they had a T-score of ≤−1.5 plus at least two prevalent vertebral fractures [41]. When calculating the annual hip fracture rate in women receiving a placebo during those two clinical trials, the rate was 0.37 % and 0.76 % respectively. If the threshold DXR T-score ≤−2.5 is applied for all women in our material, the sensitivity to find hip fractures would be 0.81 and the specificity 0.79. However, in guidelines treatment is normally recommended for the 55–85-year age group. Used in that age group, the threshold DXR T-score ≤−2.5 would result in a sensitivity of 0.76 and a specificity of 0.72, with an annual fracture rate of 1.6 %. That rate is clearly higher than those observed in most pivotal fracture trials. This higher fracture incidence illustrates the difference in fracture incidence between clinical cohorts and that of clinical trials with selected subjects. It can therefore be discussed whether treatment should be given at higher DXR T-scores than <−2.5 in order to give treatment at the same fracture risk level as those shown to be efficient in the clinical trials. However, if the threshold were based on annual hip fracture rates similar to those observed in the placebo group of the HORIZON trial (0.76 %) [41], it would result in a severely reduced specificity. Women aged 55 to 85 would then receive treatment already at DXR T-scores of ≤−0.59, resulting in a sensitivity of 0.97, but with the poor specificity of 0.23. This would lead to substantially higher numbers needed to treat in order to avoid one hip fracture.

Defining the threshold level for men is more challenging. At DXR T-score <−2.5 the sensitivity for all men >40 years is only 0.36 and specificity 0.93, with an annual fracture rate of 2.0 %. Applying a threshold of DXR T-score <−2.5 would therefore imply severe undertreatment, excluding most men at risk. In men the threshold value of osteopenia seems more reasonable when applied to the 55–85-year age group, resulting in a sensitivity of 0.52 and specificity of 0.76, with an annual hip fracture rate of 0.81 %. However, there were only 20 hip fractures in that age group, so the threshold for treatment in men when using DXR cannot be robustly estimated.

### Study limitations

The radiographs used in this study were not intentionally intended for DXR analysis. Only 41 % of the retrieved radiographs were considered suitable for analysis with DXR. This percentage might appear small, but one has to consider that all radiographs with plaster, external fixation or fractures that affected the metacarpals (i.e. measurement regions) were excluded. When retrieving radiographs from the digital archives, examinations of fingers or carpal bones were also included as they carry the same examination code as a radiograph of the hand. Such radiographs could not be included as they do not fully depict the metacarpal bones.

In some included cases, the radiographs considered acceptable for DXR analysis were actually slightly suboptimal because they were not obtained according to the DXR protocol. This source of error might affect DXR’s predictive value in this study.

According to population registers from Statistics Sweden, 78 % of the population in Stockholm was born in Sweden, 15 % in other Caucasian countries, 2.6 % in Asian countries, 2.2 % in African countries and 1.7 % in Hispanic countries. Due to the retrospective data collection of radiographs only, there was no information on patient race, body mass index or menopause age. Neither were there records on prior or subsequent treatment for osteoporosis or other diseases. This could influence the predictive value found in this study. If a great proportion of our material had treatment with cytostatics or corticosteroids, this would lead to an overestimation of the predictive value, but on the other hand osteoporosis treatment would lead to an underestimation.

The DXR analysis is intended for use on the non-dominant hand. As there was no patient record regarding hand dominance, the left hand was analysed in all patients. Approximately 10 % of the population has a left hand dominance [42]. This is a source of error, probably resulting in slightly higher DXR-BMD in left-dominant individuals, which could lead to a slight underestimation of DXR’s ability to predict future hip fractures.

The use of The Swedish National Patient Register and National Cause of Death Register minimised the risk of loss to follow-up. This is a major strength of this study due to the high quality of the registers with 99 % completion [43]. Unfortunately, we were not able to identify whether any of the patients had been exposed to high-energy trauma that could have caused their hip fracture. This is a limitation of our study as non-fragility fractures might have been included in the fracture group. However, this effect is believed to be minimal, as patients with hip fractures were on average significantly older than those without fracture and had lower DXR-BMD. Furthermore, included non-fragility, i.e. high-energy trauma, hip fractures would most likely affect DXR’s predictive ability unfavorably, resulting in an underestimation of the method’s predictive value.

In conclusion, hip fractures can be predicted in both women and men by DXR analysis of clinical wrist and hand radiographs obtained at emergency hospitals. DXR may therefore be useful to identify patients with increased hip fracture risk already at the emergency department and might provide an alternative where access to central BMD measurements is limited. However, further studies are warranted to determine DXR’s ability in a normal population.

## Abbreviations and acronyms

DXR: Digital X-ray radiogrammetry
QCT: Quantitative computed tomography
DXA: Dual-energy X-ray absorptiometry
BMD: Bone mineral density
T-score: Standard deviations above or below the mean for a healthy young adult of the same sex and ethnicity as the patient
Z-score: The number of standard deviations above or below the mean for the patient’s age, sex and ethnicity
HR: Hazard ratio
SD: Standard deviation
ROC: Receiver-operating characteristic
AUC: Area under the curve
ICD-10: International Statistical Classification of Diseases and Related Health Problems, 10th Revision
